# Visualization of aquaionic splitting *via* iron corrosion

**DOI:** 10.1038/s41598-020-58707-y

**Published:** 2020-02-03

**Authors:** Shuntaro Murakami, Lihua Zhang, Seiichi Watanabe

**Affiliations:** 1 Graduate School of Engineering, Hokkaido University, N13, W8, Kita-ku, Sapporo, Hokkaido 060-8628 Japan; 2 Faculty of Engineering, Hokkaido University, N13, W8, Kita-ku, Sapporo, Hokkaido 060-8628 Japan

**Keywords:** Chemical engineering, Corrosion

## Abstract

We report a water decomposition mode called ‘Aquaionic Splitting (AiS)’ by means of iron corrosion in aqueous solution. In this paper, we investigated the phenomenon by controlling the reaction between iron and water. A pseudo-sacrificial protection method with oil paint was employed to select the anode and cathode formation locations that govern iron corrosion. Then, the AiS reaction was visualized by using BTB solution, whose colour corresponds to pH, to produce colour patterning that corresponds to the aquaion distribution. It has become clear that water can be selectively separated into protons and hydroxide ions by corrosion control treatment. In this vein, the diffusion coefficient of protons was estimated by using the colour patterning of BTB solution that accompanies iron corrosion, and aquaion distribution was then computer simulated by solving the diffusion equation.

## Introduction

In metal corrosion in an aqueous solution, for example, iron corrosion in water, the formation of oxides and hydroxides is accompanied by the generation of local anodes and cathodes on the surface of a base material^1–4^. Simultaneously, a substantial decomposition reaction of water occurs in conjunction with the corrosion reaction. It is well known that water electrolysis generates hydrogen gas and oxygen gas by the complete decomposition of water^5^ *(H*_2_*O* → *H*_2_ + *1/*2 *O*_2_) and that the generation of and hydroxide ions from water, that is, the aquaionic splitting (AiS) *(H*_2_*O* → *H*^+^  + *OH*^−^) reaction of water, takes place as a cooperative reaction. In addition, it is noteworthy that the AiS reaction occurs at an energy of 0.83 eV as calculated by HSC Chemistry software (Outokumpu Research Oy, Pori, Finland), which is lower than the energy (1.23 eV) required for the complete electrolysis of water. Accordingly, we found similarities between the water decomposition reaction of electrolysis and the AiS reaction associated with iron corrosion.

Generally, it is difficult to corrode a whole piece of iron in aqua because the oxidation/reduction reaction proceeds with random formation of the anode and the cathode. However, to observe a substantial water decomposition reaction, it is necessary to clearly distinguish and control the places where the oxidation and reduction reactions occur. Anticorrosion is then expected to control the corrosion location, and the plating method, which is a sacrificial anticorrosion method, is a well-known example^6,7^. We focused on sacrificial protection in the present study. However, in addition to the need for preparation of the electrolyte in the plating method^8^, there are concerns about the introduction of impurities because other metals are used^9^. Therefore, we adopted a facile corrosion protection method using a conductive oil-based paint, which is expected to have a pseudo-sacrificial effect, in addition to other similar proposed methods^10,11^.

By controlling the corrosion areas locally, we tried to observe the AiS reaction of water accompanying iron corrosion, which is visualized by using a bromothymol blue (BTB, C_24_H_28_Br_2_O_5_S) solution^12,13^ whose colour corresponds to pH. In this study, diffusion coefficient measurement of ion species is also performed with the unprecedented simple method of using the colour patterning formed by the BTB solution and a computer simulation of 2-dimensional aquaion diffusion *via* AiS. In general, the methods of measuring the diffusion coefficient of ion species in a dilute solution or gel include the use of a radioactive tracer^14^, the holographic laser method^15^, the nuclear magnetic resonance method^16^ and the fluorescence method^17^. However, in these methods, it is not an easy task to detect the diffusion of protons in an aqueous corrosive environment, and a dedicated device is required.

## Results and Discussion

### Visualization of AiS reaction via iron corrosion in BTB agar gel

**Figure 1:**
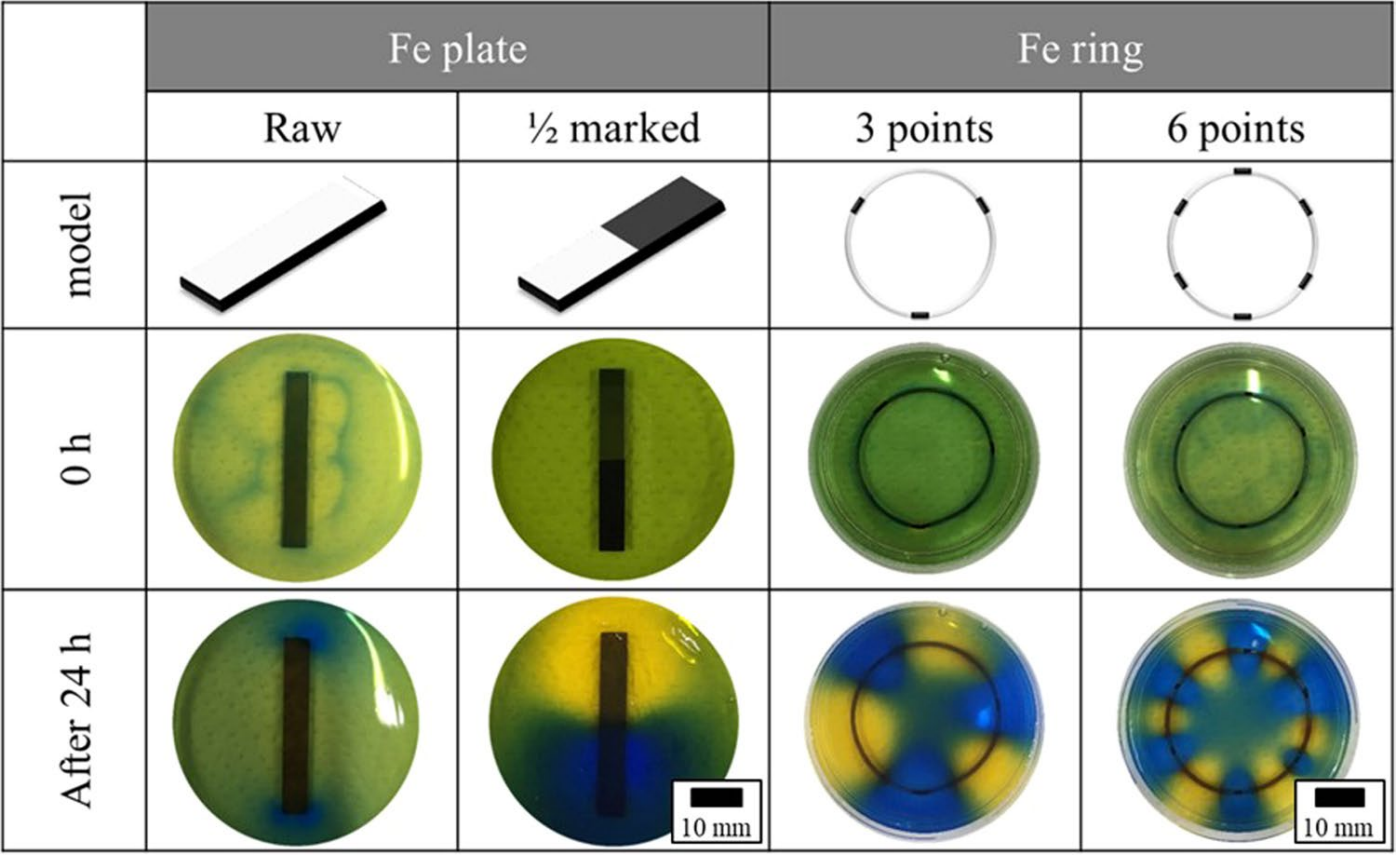
Visualization of aquaionic splitting with the alkaline (blue) and acidic (yellow) distribution via Fe corrosion in BTB agar gel from 0 to 24 hours.

Figure 1 shows the observed results of the AiS reaction in BTB agar gel using plate- and ring-like Fe samples. Compared with the untreated sample, in any of the samples subjected to anticorrosion treatment, the colour of the agar gel around the area where the paint is applied changes from green to blue, while the colour of the region around the area without paint changes from green to yellow, indicating that AiS colour patterning was formed by iron corrosion. It turns out that the yellowish region of the BTB agar around the corrosion area was acidic, while the bluish region around the anticorrosion area was alkaline, suggesting that proton is generated in the area where corrosion progresses, as the pH is lowered, and that hydroxide ion is generated in the area where corrosion is prevented, as the pH is increased, i.e., where the AiS reaction *(H*_2_*O* → *H*^+^ + *OH*^−^) occurs.

**Figure 2:**
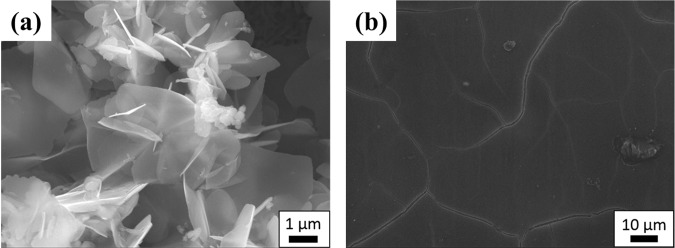
SEM image of the Fe sample after corrosion for 24 hours. **(a)** The corroded site of Fe. **(b)** The anticorrosion site of Fe.

Therefore, it becomes clear that controlling the corrosion site of iron can enhance the AiS reaction with the aid of visualization of the aquaion distribution that accompanies iron corrosion by forming a colour pattern using BTB agar. Furthermore, the corroded, anodic part was confirmed by the naked eye, and the presence of a corrosion product was observed by SEM (Fig. 2).

### Detection of protons generation

**Figure 3:**
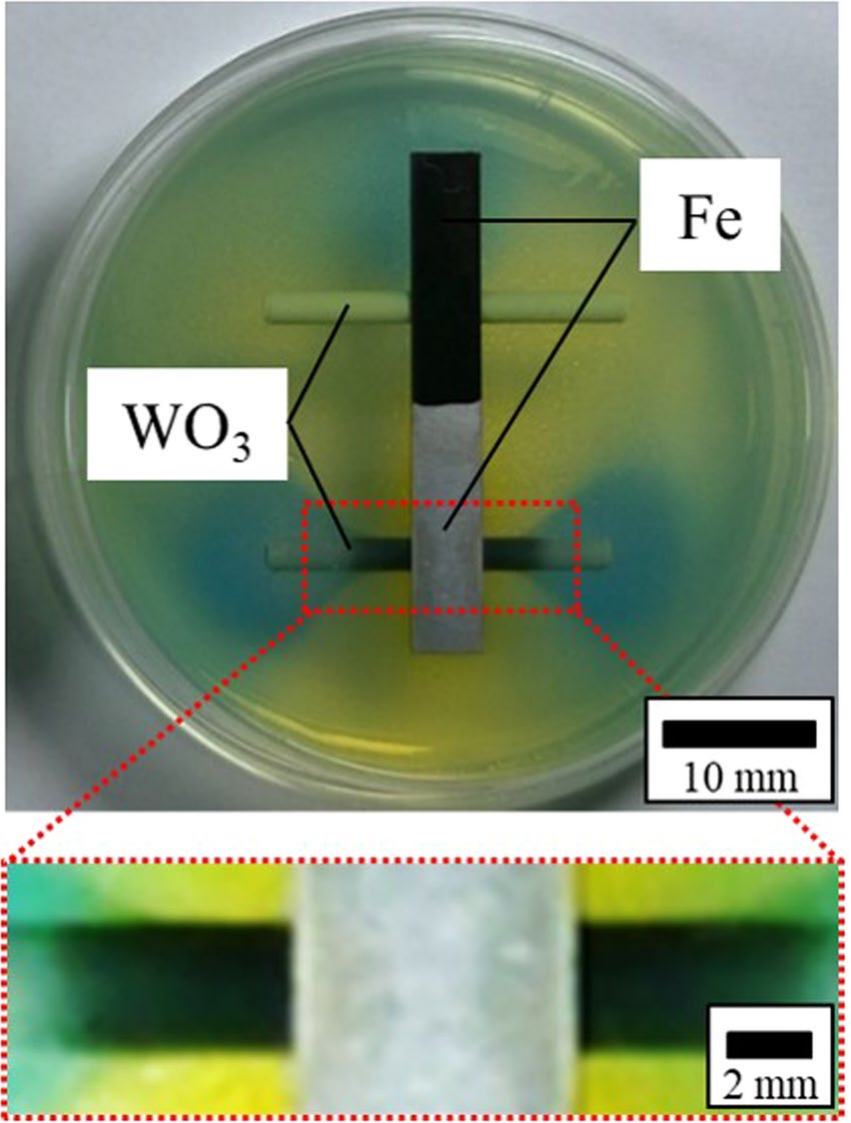
Image of Fe plate and WO_3_ rod inserted in BTB agar gel and magnified image of WO_3_ rod (red square).

By colour patterning using BTB, the diffusion of hydroxide ions and protons can be predicted from the anti-corrosion/corrosion points, respectively. In Fig. 3, one can observe hydrogen generation during iron corrosion with rod-like WO_3,_ which is used as a proton detection probe (see the Methods section). The image enclosed by the red square is a magnified image of the WO_3_ rod. The colour of the rod-like WO_3_ in contact with the anticorrosion iron plate did not change, while the colour of WO_3_ in contact with the corroded part changed to dark blue.

It was evident that tungsten oxide (WO_3_) was changed to tungsten bronze (HWO_3_) by the chromic reaction^18,19^, indicating the release of protons and electrons from the corroded part of iron according to reaction (6) in the method section. Therefore, it was confirmed that the formation of the aquaion distribution from the anode side is due to the generation of protons and electrons.

### Considerations regarding AiS reactions associated with iron corrosion

The results suggested that the corrosion site acts as an anode and the anticorrosion site as a cathode. Protons and hydroxide ions were generated and diffused from each site. In addition, since the experiment is performed at room temperature and no special operation is needed, the reaction proceeds spontaneously, i.e., the AiS reaction proceeds when the free energy change *ΔG* takes a negative value. In the present study, the reaction equation was considered from the viewpoint of free energy using HSC Chemistry Software, which is a free energy calculation software. The corresponding temperature of 25 °C was assumed in the calculation.

The SEM image of the sample surface after the AiS reaction is shown in Fig. 2. It was confirmed that the corrosion product was present at the corrosion site but was not found at the anticorrosion site. Although the corrosion products of iron are varied, the final product was considered to be iron hydroxide (Fe(OH)_3_)^20^, as shown SEM observation in Fig. 2(a), XRD pattern and XPS spectra in Figs. S3 and S4, respectively.

#### The reaction at the corrosion site (anode)

It is expected that this site behaves in almost the same manner as general iron corrosion in aqua. The corrosion reaction proceeds in two steps. First, when the sample is inserted into water, an iron leaching reaction (1) occurs, and then, some of the electrons move to the cathode side.

1 $$Fe=F{e}^{2+}+2{e}^{-}(\Delta G=-\,91.5\,kJ/mol)$$

Second, reaction (2) occurs, with the generation of Fe(OH)_3_ and the release of protons:

2 $$F{e}^{2+}+1/4\,{O}_{2}+5/2\,{H}_{2}O=Fe{(OH)}_{3}+2{H}^{+}(\varDelta G=-\,24.6\,kJ/mol)$$

The net reaction is as follows:

3 $$Fe+1/4\,{O}_{2}+5/2\,{H}_{2}O=Fe{(OH)}_{3}+2{H}^{+}+2{e}^{-}(\varDelta G=-\,116.1\,kJ/mol)$$

This reaction formula is well known^21^ and agrees with the result of protons detection by tungsten oxide probe.

On the anode side, the pH decreases mainly according to these reactions, and the colour of BTB agar gel changes to yellow. Although a reaction in which divalent iron hydroxide is generated as an intermediate may also occur (*Fe*^2+^ *+* *2OH*^−^ = *Fe(OH)*_2_), it is not explicitly expressed in the above AiS reaction.

#### The reaction at the anticorrosion site (cathode)

Reaction (1) on the anode side generates divalent iron ions and electrons. Then, the iron ion transition as described above is the main corrosion reaction, and electrons transfer to the cathode. A rise in pH is confirmed on the cathode side as the colour of BTB agar gel changes to blue. This is because reaction (4) predominantly occurs, as follows:

4 $${H}_{2}O+1/2\,{O}_{2}(g)+2{e}^{-}=2O{H}^{-}(\varDelta G=-\,77.4\,kJ/mol)$$

In general, the corrosion reaction of iron in water proceeds by reaction (3) and (4), forming a cathode and anode randomly at the iron-water interface. However, by controlling the iron corrosion sites, the AiS reaction *(H*_2_*O* → *H*^+^ + *OH*^−^) can be performed separately.

### Evaluation of the diffusion coefficient of proton

**Figure 4:**
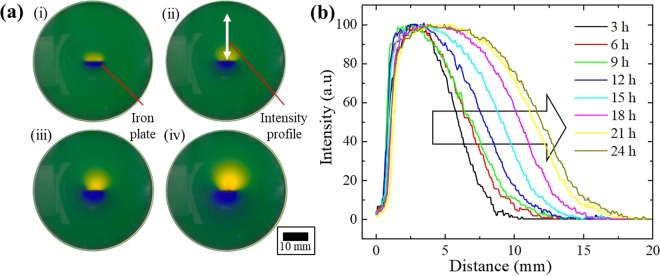
(**a**) Image of BTB agar gel with Fe plate inserted for 24 hours (i) 0.5 hours (ii) 3 hours (iii) 12 hours (iv) 24 hours. **(b)** The colour intensity spectra for 3–24 hours.

An image of the observed BTB agar gel sample for the measurement of the diffusion coefficient of protons is shown in Fig. 4(a). A plate-like iron sample is placed at the centre of a petri dish. It is shown that the yellow area spreads with the passage of time. Image analysis was performed linearly from the centre of the iron sample in each image using a Gatan Digital Micrograph (Gatan Microscopy Suite, USA) to obtain a numerical colour intensity. Figure 4(b) shows a measurement in which the vertical axis is the colour tone intensity and the horizontal axis is the diffusion distance. The yellowish colour intensity of the BTB agar gel is extracted. From this result, it is numerically shown that the diffusion of protons proceeds with time.

**Figure 5:**
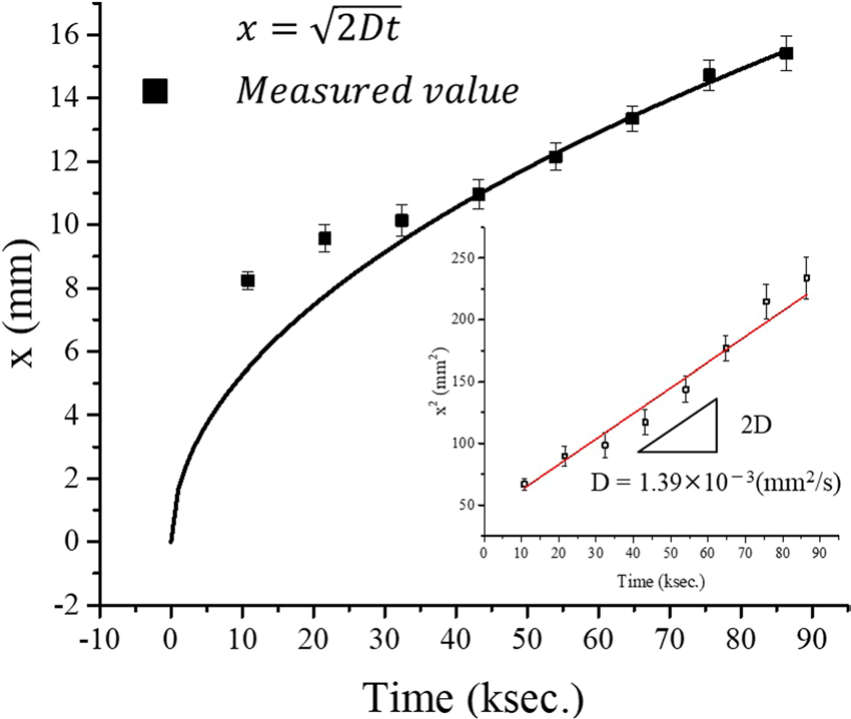
A diffusion distance and time graph during AiS reaction. The black line follows the equation ${\rm{x}}\,=\,\sqrt{2Dt}$ using D = 1.39 × 10^−3^(mm^2^/s). The inset shows a graph for calculating the diffusion coefficient D of protons. The red line is a fitting curve.

The diffusion distance *x* of protons evaluated from the graph is summarized in Table 1. These numerical values are plotted in Fig. 5. The vertical axis is the diffusion distance *x*, and the horizontal axis is the time *t*. The inset is a graph in which the vertical axis is the square of the diffusion distance *x* and the horizontal axis is time *t*, and the red line is obtained using linear fitting. If the diffusion process is considered to be one-dimensional, the diffusion distance is calculated by Eq. (5).

5 $${x}\,=\,\sqrt{2{Dt}}$$

*x: Diffusion distance, D: diffusion coefficient, t: time*

**Table 1:** The diffusion distance of protons from 10% intensity in Fig. 4(b).

Time (ksec.)	x (mm)
0	0.0
10.8	8.2
21.6	9.5
32.4	9.9
43.2	10.8
54.0	12.0
64.8	13.3
75.6	14.6
86.4	15.3

From the inset in Fig. 5, D = 1.39 × 10^−3^(mm^2^/s) was obtained as the value of the diffusion coefficient *D*, using the slope (*2D*) of the fitting line. The black line in Fig. 5 is a curve drawn using the resultant value. Although there is some deviation, it almost follows Eq. (5). Additionally, according to the literature, the diffusion coefficients of protons in water and gel are close to D_water_ = 8.24 × 10^–3^(mm^2^/s), obtained by scaling the water interatomic potential model^22^, and D_gel_ = 6.22 × 10^–3^(mm^2^/s), obtained from PFGSE-NMR spectroscopy^23^, respectively. Although the measured values in the present study yield slightly lower values than the above, the diffusion coefficient of protons can be estimated using the BTB agar gel method.

### Simulation for AiS reaction by colour patterning

**Figure 6:**
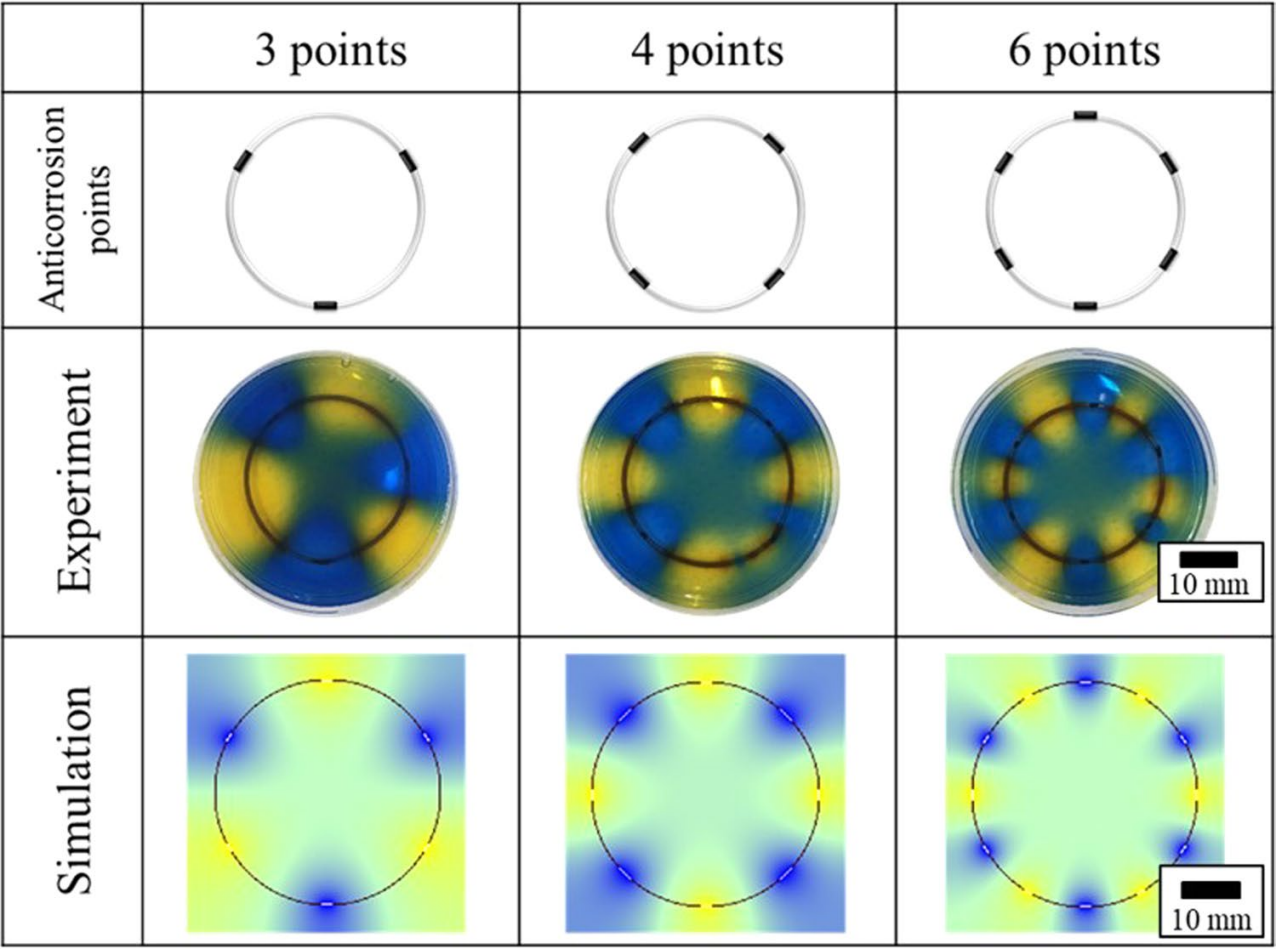
Image of observation and simulation for ion concentration distribution during AiS reaction (24 hours).

In the computer simulation described in the methods section, the diffusion coefficient D = 1.39 × 10^−3^(mm^2^/s) obtained in the previous section was used. Figure 6 shows the experimentally observed results (24 hours) for ring-shaped iron samples and the simulation results (24 hours). The simulation results and the experimental results were consistent with each other. In addition, the results were consistent even if the generation sites of protons and hydroxide ions were increased. As a consequence, the diffusion of protons and hydroxide ions accompanying the AiS reaction due to iron corrosion can be described by an ordinal diffusion equation.

## Conclusions

In this study, we controlled the corrosion site of iron arbitrarily and analysed the ion separation and aquaion distribution in water accompanying the corrosion. As a result of the anticorrosion treatment, the area around the anticorrosion site became alkaline, and the area around the corrosion site became acidic. Therefore, it was found to be possible to cause the AiS reaction by facile control of the corrosion sites. Moreover, it turned out that the formation of colour patterning accompanying the AiS reaction and the visualization of corrosion are possible by using BTB solution, whose colour corresponds to acidic or alkaline pH. In addition to estimation of the diffusion coefficient for the aquaion by using colour patterning, it is possible to carry out corrosion analysis in an unprecedented method by computer simulation of diffusion. The above facts can be used for the corrosion analysis of other metals. Although the details will be reported in the near future by the present authors, the actual pH in anodic and cathodic regions under AiS in neutral water can also be monitored and the typical pH values of these regions are ~4 and ~10, respectively, after 48 hours of the AiS reaction.

## Methods

### Materials and pretreatment

**Figure 7:**
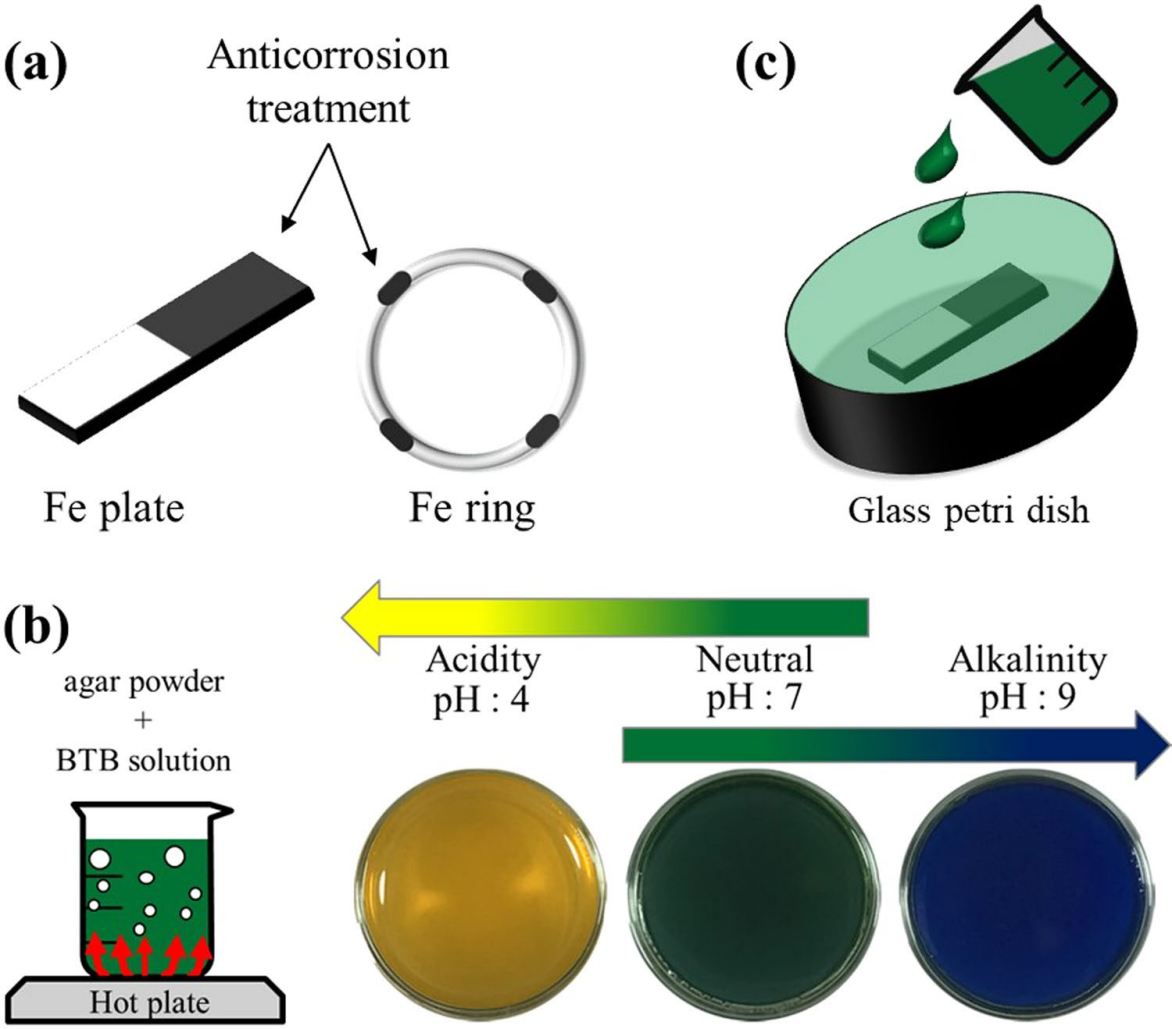
Visualization of the ion concentration distribution with (**a**) Fe samples coated with oil-based paint as an anticorrosion treatment. (**b**) Preparation of BTB agar gel and its coloration: green (neutral), yellow (acidic), and blue (alkaline). (**c**) Observation of BTB agar gel containing Fe samples.

The sample material was an Fe plate (99.95%) with dimensions of 35 × 5 × 0.5 mm (Nilaco, Tokyo, Japan) and an Fe ring with a diameter of 30 mm (Nilaco, Tokyo, Japan). Ultrasonic cleaning of the samples was performed for 5 minutes with ethanol, followed by 5 minutes with distilled water, and the samples were dried. An oil-based paint (Pilot Japan) was used to coat appropriate sites on the surface of the sample as an anticorrosion treatment (Fig. 7(a)).

### Visualization of the AiS accompanying iron corrosion

Since a substantial water decomposition reaction is expected to be accompanied by a pH change, visualization was performed by using bromothymol blue (BTB) solution (C_24_H_28_Br_2_O_5_S, molar mass = 0.04 W/V%, SHOWA, 6.25%). A BTB agar solution was prepared by boiling 20 ml of BTB solution mixed with 5 g of agar (Hayashi Pure Chemical Ind., Ltd.) and 80 ml of purified water at 100 °C (Fig. 7(b)). The prepared BTB solution gel colour will turn from green to yellow and blue to denote acidic pH (~6.0) and alkaline pH (7.6~)^24^. Then, the plate and ring Fe samples were immersed in the BTB agar gel, and the colour change of the BTB agar gel was observed (Fig. 7(c)).

### Hydrogen detection

**Figure 8:**
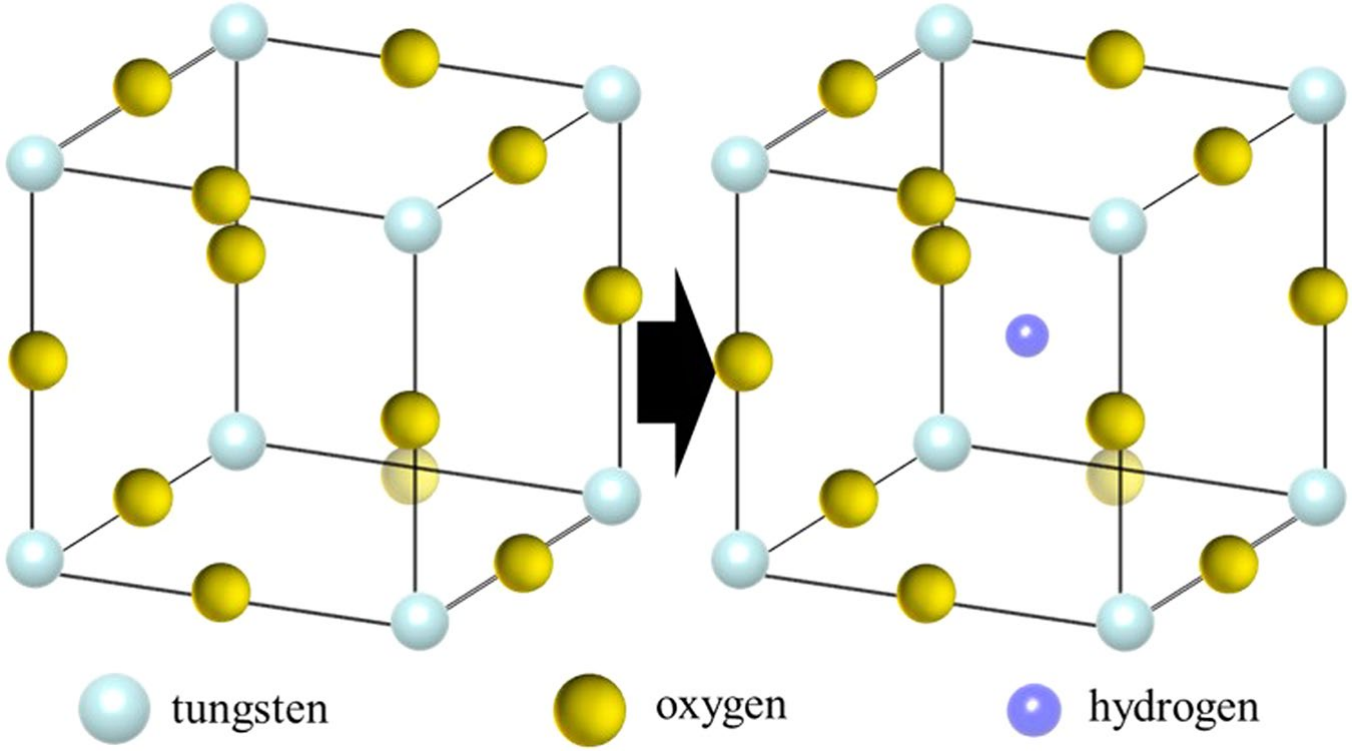
Drawings of the crystal lattice of WO_3_ (left) and HWO_3_ (right).

Tungsten oxide (WO_3_) was used as a hydrogen detection probe. Tungsten oxide is a yellow substance and has a perovskite structure (ABO_3_) with missing A site^25,26^. In the crystal lattice, tungsten ions exist in a hexavalent state (W^6+^), thereby exhibiting a yellow colour. Tungsten oxide changes to tungsten bronze^27^ by reacting with protons and electrons, as shown in the following reaction formula (6) and in Fig. 8. At that time, protons enter the A site of tungsten oxide, and the tungsten ions are reduced by electrons to change to pentavalent ions (W^5+^). This is a chromic reaction, and since tungsten bronze is blue in colour, it is possible to detect hydrogen generation by observing a change in colour.

6 $${W}{{O}}_{3}+{{H}}^{+}+{{e}}^{-}\to {HW}{{O}}_{3}$$

Rod-like tungsten oxide was used for the experiment. It was prepared by heating rod-like metallic tungsten (φ1 mm) under atmospheric conditions in an electric muffle furnace at 800 °C for 1 hour. An as-prepared iron plate was inserted into an agar gel-filled glass petri dish. Then, rod-like tungsten oxide was placed on the corrosion site and the anticorrosion site. Thus, the change in colour of tungsten oxide, which is a hydrogen detection material, was confirmed (see Fig. 3).

### Diffusion coefficient measurement

In the centre of a glass petri dish containing BTB agar gel, a plate-like iron sample was placed sideways and perpendicular to the petri dish. Anticorrosion treatment was applied to only one side of the Fe plate. The results in the glass dish were observed with a video camera. After observation, the colour of the BTB agar gel was subjected to linear image processing from the centre of the iron sample using a Gatan Digital Micrograph (Gatan Microscopy Suite, USA) to graph the intensity of the colour tone by numerical values. From the graph, the diffusion distance of protons is obtained as follows.

Draw a straight line at 10% on the graph. The intensity range is 0 to 100, but 10% or less is rounded off to reduce the error. The distance between the intersection of the straight line and the intensity curve is recorded from the values on the graph.

### Computer simulation of AiS

**Figure 9:**
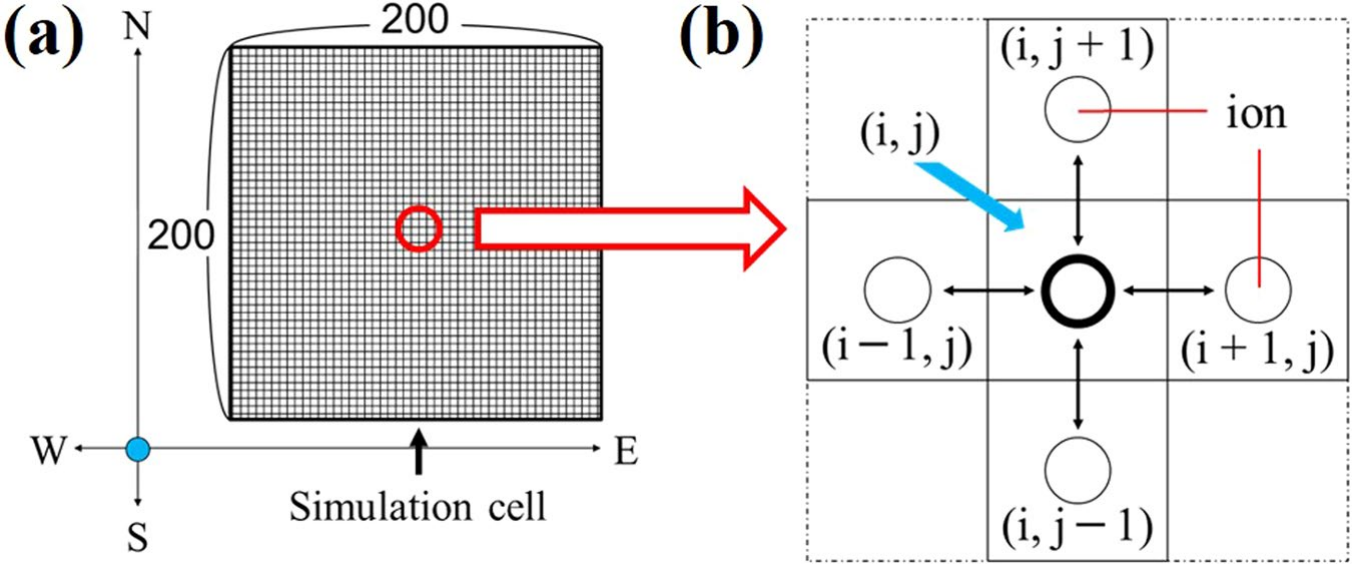
(**a**) Illustration of the calculation area for the computer simulation. (**b**) Is the enlarged image.

As shown in Fig. 9(a), the calculation area is a square divided into 200 × 200 cells. The following conditions were given to each cell.

(i) Liquid phase (agar gel)
(ii) Ring-shaped iron sample
(iii) Ion generation site (proton, hydroxide ion)

The anticorrosion site corresponded to the hydroxide ion generation point, whereas the corrosion site corresponded to the proton generation point. In addition, the concentration of each ion species was set to +1 at the proton generation point and −1 at the hydroxide ion generation point. The simulation was carried out by numerically solving Fick’s law.

Figure 9(b) is an enlarged view of the cells. Assuming that the diffusion of ion species follows Fick’s first law (7), the time change of the ion amount of each cell was calculated. The ion fluxes flowing into and out of the central cell (*i, j*) from the top, bottom, left and right cells ((*i, j* + 1), (*i, j* − 1) (*i* + 1, *j*) (*i* − 1, *j*)) were calculated by the finite difference method. The top, bottom, left, and right correspond to the N-S axis and W-E axis, respectively. The fluxes between cells are then expressed as:

7 $$\begin{array}{rcl}{J}_{k} & = & -D\frac{dc}{d{x}_{k}}\\ {J}_{N} & = & D\frac{{C}_{i,j+1}^{t}-{C}_{i,j}^{t}}{\varDelta y}{\Delta }x\cdot {\Delta }t,{J}_{S}=D\frac{{C}_{i,j-1}^{t}-{C}_{i,j}^{t}}{{\Delta }y}{\Delta }x\cdot {\Delta }t\\ {J}_{W} & = & D\frac{{C}_{i-1,j}^{t}-{C}_{i,j}^{t}}{{\Delta }x}{\Delta }y\cdot {\Delta }t,{J}_{E}=D\frac{{C}_{i+1,j}^{t}-{C}_{i,j}^{t}}{{\Delta }x}{\Delta }y\cdot {\Delta }t\end{array}$$

*J: ion flux, D: diffusion coefficient, C: concentration*

The sum of the ion amounts flowing into and out of the central cell (*i, j*) in *Δt* is

$$\left({C}_{i,j}^{t+{\Delta }t}-{C}_{i,j}^{t}\right){\Delta }x\cdot {\Delta }y=\left({J}_{N}+{J}_{S}+{J}_{W}+{J}_{E}\right)$$

From this equation, the concentration after *Δt* of the central cell is calculated as follows.

$${C}_{i,j}^{t+{\Delta }t}={C}_{i,j}^{t}+\frac{({J}_{N}+{J}_{S}+{J}_{W}+{J}_{E})}{{\Delta }x\cdot {\Delta }y}$$

The change in ion amount was calculated for each cell for *Δt*. We also used the zero-flux boundary condition on the outer cells.

## Supplementary information

Supplementary information.
