# Supplementary material for: Visualization of aquaionic splitting via iron corrosion
Source: Sci Rep. 2020 Feb 3;10:1726. doi: 10.1038/s41598-020-58707-y (PMC6997388; doi:10.1038/s41598-020-58707-y)
Supplement: Supplementary file 1 — Supplementary information. [file 41598_2020_58707_MOESM1_ESM.docx]

**Supplementary Information**

**Visualization of aquaionic splitting *via* iron corrosion**

Shuntaro Murakami^1^, Lihua Zhang^2^, Seiichi Watanabe^2^

^1^Graduate School of Engineering, Hokkaido University, N13, W8, Kita-ku, Sapporo, Hokkaido, 060-8628, Japan. ^2^Faculty of Engineering, Hokkaido University, N13, W8, Kita-ku, Sapporo, Hokkaido, 060-8628, Japan. Corresponding and requests for materials should be addressed to S.W. (email:sw004@eng.hokudai.ac.jp)


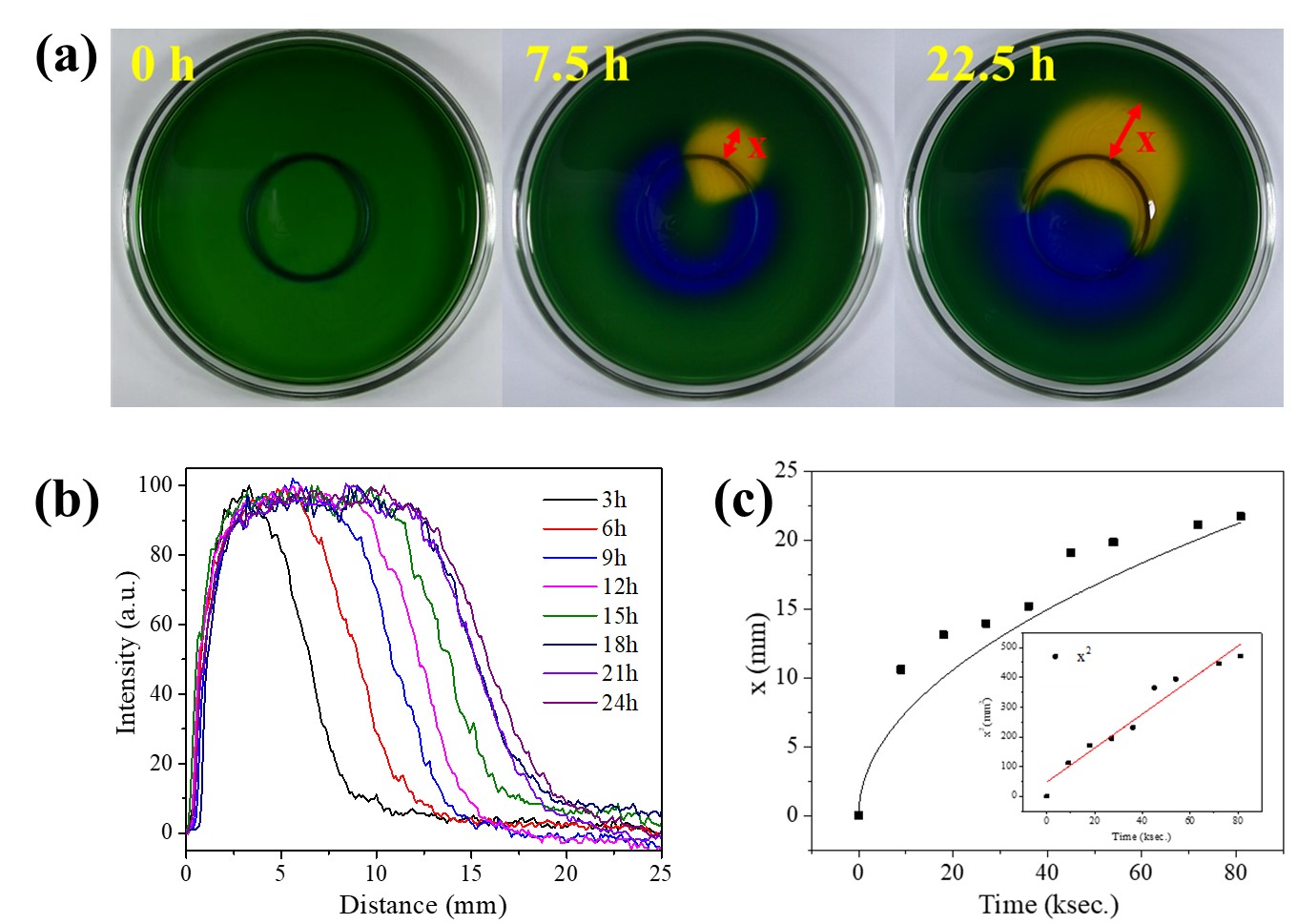


Figure S1. (a) Image of BTB agar gel inserted Fe ring for 0 h (left), 7.5 h (center), 22.5 h (right) (b) The color intensity spectra for 3-24 hours. (c) A diffusion distance and time graph during AiS reaction. The black line is following equation (5) using D = 2.1×10^-3^ (mm^2^/s). Inset is a graph for calculation the diffusion coefficient D of hydrogen ion. The red line is a fitting one.


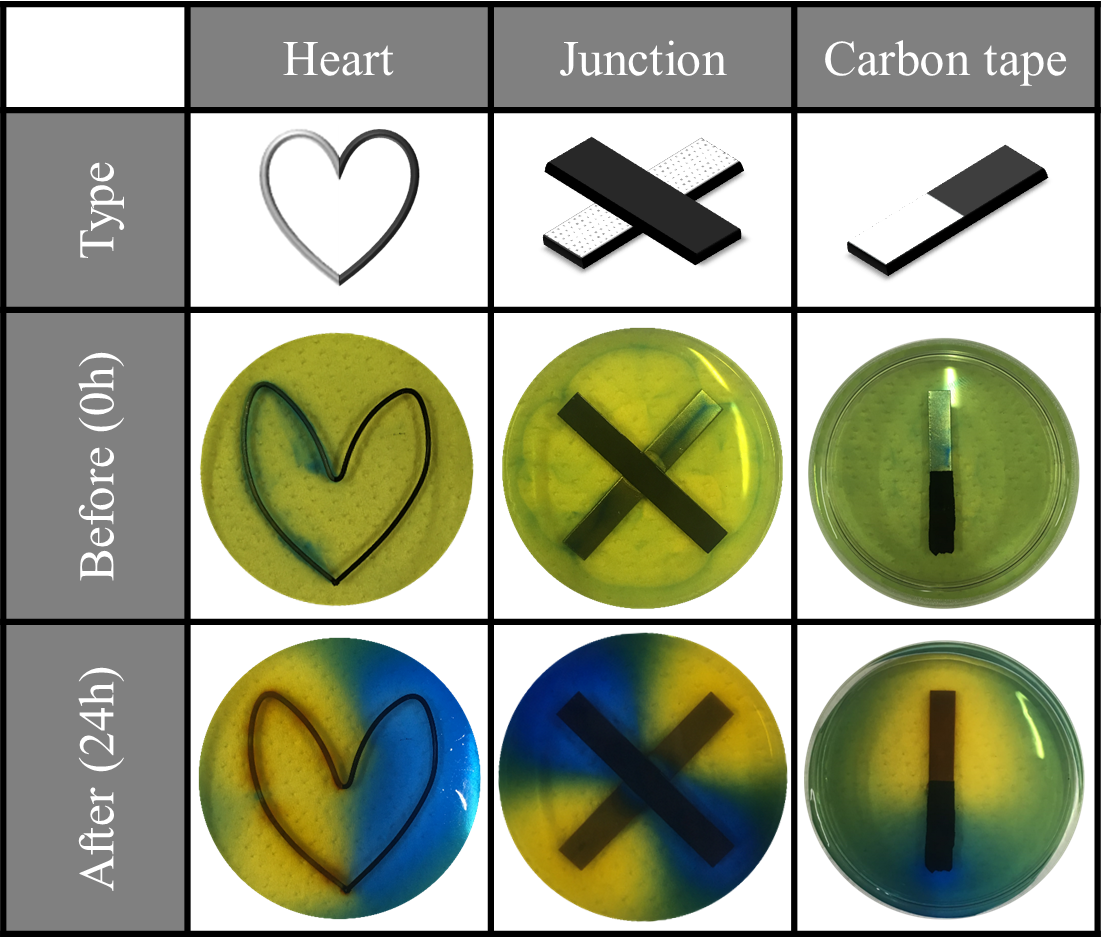


Figure S2. Visualization and color patterning of aquaionic splitting with the alkaline (blue) and acidic (yellow) distribution *via* Fe corrosion in BTB agar gel from 0 hour to 24 hours. The left column is a heart-like shaped iron wire, the center column is joining raw Fe plate and anticorroded Fe plate with oil paint, the right column is by the anticorroded treatment with a conductive carbon tape instead of painting.


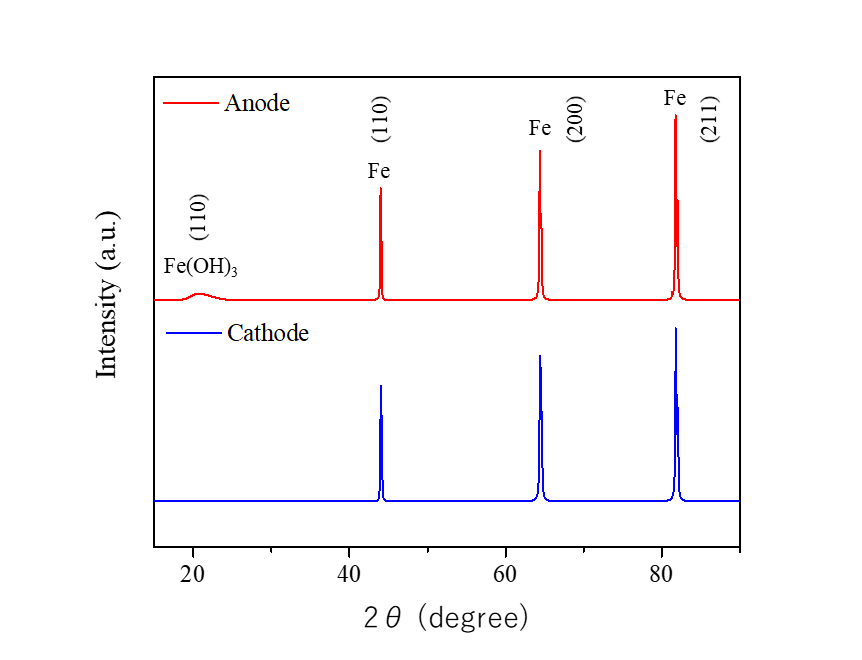


Figure S3. XRD diffraction pattern of the anode and cathode part of the Fe sample after AiS reaction for 24h.


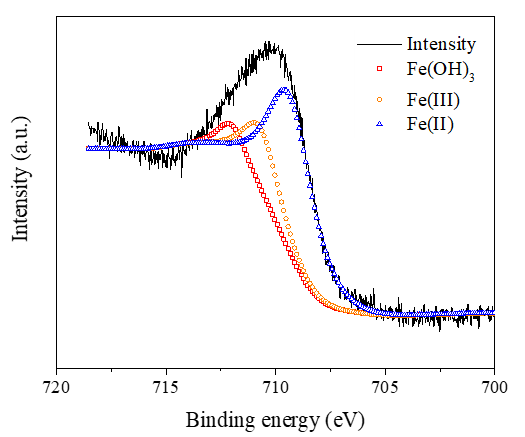


Figure S4. XPS spectra of the Fe 2p_3/2_ of the anode part after AiS reaction for 24 h.
